# World Health Organisation’s (WHO) Study on Global Ageing and Adult Health (SAGE)

**DOI:** 10.1186/1753-6561-7-S4-S1

**Published:** 2013-08-23

**Authors:** Somnath Chatterji

**Affiliations:** 1 Multi-Country Studies Unit, World Health Organisation (WHO), Geneva, Switzerland

## 

The numbers of older adults are growing rapidly globally with the rate of increase larger in less developed countries. As life expectancies increase around the world, it is a priority to determine if more of the extra years being added are healthy years or years that are likely to be spent in poor health.

The Study on Global Ageing and Adult Health (SAGE) of the World Health Organisation (WHO) (http://www.who.int/healthinfo/sage) is a longitudinal nationally representative household survey that includes respondents 50 years and older with a smaller, comparative cohort of adults aged 18-49 years in China, Ghana, India, Mexico, Russia and South Africa with a sample size of over 40,000 respondents selected using a multi-stage cluster design. Additionally, eight health and demographic surveillance sites (HDSS) in Bangladesh, Ghana, India, Indonesia, Kenya, South Africa, Tanzania and Viet Nam with an additional combined sample size of over 45,000 respondents are a part of SAGE. The Collaborative Research on Ageing in Europe (COURAGE) project has also used SAGE methods and instruments to collect data in Finland, Poland and Spain.

The objective of SAGE is to improve the empirical understanding of the health and well-being of older adults through provision of reliable, valid and cross-nationally comparable data over time on key outcomes and determinants. Wave 0 was completed in 2004 with Wave 1 finalised in 2010. Wave 2 of SAGE is planned for later in 2013 and Wave 3 in 2015. The biomarker component of SAGE includes performance tests and the collection of dried blood spots in Wave 1. Dried Blood Spot (DBS) samples have been collected from approximately 40,000 respondents and stored. The assays for haemoglobin, glycosylated haemoglobin (HbA1c), high sensitivity C-reactive protein (hsCRP), Epstein Barr Virus (EBV) and HIV are being carried out initially with additional assays planned for the future. Future waves of SAGE will consider the collection of DNA samples.

First results from SAGE reveal significant declines in health over the life span with female and poorer respondents being in worse health at all ages. Chronic health conditions are extremely prevalent. Risk factors such as tobacco use, inadequate physical activity, obesity and hypertension are all very common. Poor health is associated with declining subjective well-being and shrinking social networks.

This suggests that there is little evidence of healthy ageing in these populations and that a concerted response will be required from health systems to address the needs of this rapidly growing segment of the population.

